# Properties of humic acids depending on the land use in different parts of Slovakia

**DOI:** 10.1007/s11356-021-14616-9

**Published:** 2021-06-08

**Authors:** Magdalena Banach-Szott, Bozena Debska, Erika Tobiasova

**Affiliations:** 1 Department of Biogeochemistry and Soil Science, University of Science and Technology, 6 Bernardynska St, 85-029 Bydgoszcz, Poland; 2 Department of Soil Science, Slovak University of Agriculture in Nitra, 2 Tr. A. Hlinku St, 94976 Nitra, Slovakia

**Keywords:** Soils, Elemental composition, UV-VIS, HPLC

## Abstract

Many studies report organic carbon stabilization by clay minerals, but the effects of land use and soil type on the properties of humic acids (HAs) are missing. The aim of the paper is to determine the effects of land use and soil types on the characteristics of HAs, which have a considerable influence on organic matter quality. It was hypothesised that the effect of the land use on HAs properties depends on the particular size distribution. The research was performed in three ecosystems: agricultural, forest, and meadow, located in Slovakia. From each of them, the samples of 4 soil types were taken: *Chernozem*, *Luvisol*, *Planosol*, and *Cambisol*. The soil samples were assayed for the content of total organic carbon (TOC) and the particle size distribution. HAs were extracted with the Schnitzer method and analysed for the elemental composition, spectrometric parameters in the UV-VIS range, and hydrophilic and hydrophobic properties, and the infrared spectra were produced. The research results have shown that the properties of HAs can be modified by the land use and the scope and that the direction of changes depends on the soil type. The HAs of *Chernozem* and *Luvisol* in the agri-ecosystem were identified with a higher “degree of maturity”, as reflected by atomic ratios (H/C, O/C, O/H), absorbance coefficients, and the FT-IR spectra, as compared with the HAs of the meadow and forest ecosystem. However, as for the HAs of *Cambisol*, a higher “degree of maturity” was demonstrated for the meadow ecosystem, as compared with the HAs of the agri- and forest ecosystem. The present research has clearly identified that the content of clay is the factor determining the HAs properties. Soils with a higher content of the clay fraction contain HAs with a higher “degree of maturity”.

**Supplementary Information:**

The online version contains supplementary material available at 10.1007/s11356-021-14616-9.

## Introduction

The role of organic matter (OM), including humic substances (HS), in the soil formation and development is unquestionable. OM is also an important indicator of soil fertility, and it plays an essential role in the efficiency of agriculture, the quality of the environment and global climate changes (Zech et al. 1997; Lal 2004; Schmidt et al. 2011; Tan 2014).

The content and properties of organic matter are conditioned by the physicochemical properties of the parent rock and climate conditions. However, the land use change is the most dynamic factor of soil organic matter (SOC) changes (Guo and Gifford 2002; Poeplau et al. 2011; Jonczak 2013; Viscarra-Rossel et al. 2014; Kukuls et al. 2019; Kunlanit et al. 2019). In the temperate climate zone, the capacity of soil for SOC storage increases in the following order: cropland < forest < grassland (Meersmans et al. 2008; Martin et al. 2011). Leifeld et al. (2005) and Lettens et al. (2005) claim that the forest SOC stocks can be higher than grassland stocks, and a change in use from forest to farmland results in significant declines in SOC stocks (Guo and Gifford 2002; Murty et al. 2002; Wei et al. 2014; Kunlanit et al. 2019). Wei et al. (2014) report on the largest decrease in the SOC stock in topsoil (0–30 cm) in temperate regions - 52% followed by tropical regions - 41% and boreal regions - 31% (the values are the average decrease in the SOC stock for 3 periods - ≤10, 11–50, > 50 years which lapsed since the woodland was transformed into arable land).

One of the key SOC-stabilizing mechanisms is the soil interaction with mineral particles (Sollins et al. 1996; Von Lutzow et al. 2006). Due to a strong correlation between the SOC stocks and the content of clay presented in numerous research reports, the soil texture can be a promising factor applied as the SOC storage indicator (Hassink et al. 1997; Kaiser and Guggenberger 2000; Arrouays et al. 2006; Zinn et al. 2007).

As already mentioned, one of the most important organic carbon (OC) reservoirs is the soils of grasslands and forest soils. For that reason, the meadow and forest ecosystems are a land use preventing OC losses in soils. The OC losses triggered by soil processes are gas emissions of CO_2_ and CH_4_ as well as soluble organic carbon leaching to the groundwaters. It has been demonstrated that meadow ecosystems help limiting the OC losses from soil, and they are essential for the sustainable management of that element in the environment (Minami et al. 1993; Conant et al. 2001; Mannetje 2002; Lal 2011; Kampf et al. 2016).

Many reports point to progressive decreases in HS concentrations in soils converted from forest to arable land (Spaccini et al. 2006; Gonzalez-Perez et al. 2007; Guimaraes et al. 2013; Barancikova et al. 2016; Kunlanit et al. 2019), and as claimed by Feller and Beare (1997), Watanabe et al. (2001), and Jonczak (2013), the changes in land use can alter the chemical properties of soil HS. HS (fulvic acids, HAs, and humins) are the main fractions of SOM making its specific contribution to soil fertility. HS constitute a considerable part of the resources of OC and nitrogen (N) (Lal 1994; Milori et al. 2002; Zhang et al. 2019). Those compounds take part in all the processes which occur in soil and which affect its physical, chemical, and biological properties. HS improve the soil buffering capacity, supply the plants with available micronutrients, and immobilize organic contaminants and metals (McCarthy 2001; Yamashita et al. 2008; Canellas et al. 2010; Lanyi 2010). HS also determine the soil production potential, and by participating in the global carbon cycling, they play environmental functions (Hayes and Clapp 2001; Piccolo 2001; Lal 2006). For that reason, it is important to understand the nature, composition, and the dynamics of HS.

The molecules of HAs, with a humification progress, show a growing stability, e.g., in terms of elemental composition and spectroscopic properties (Dergacheva et al. 2012; Zhang et al. 2019). For that reason, one of the basic indicators applied to evaluate the properties of HAs is the elemental composition and, determined with it, values of the H/C, O/C, and N/C atomic ratios. The numerical values of atomic ratios facilitate an approximation of the structure of the molecules of humic acids by evaluating the degree of condensation of aromatic rings (H/C ratio) and the degree of maturity (O/C, O/H, N/C ratios) (Rice and MacCarthy 1991; Fuentes et al. 2007; Canellas et al. 2010; Trubetskaya et al. 2013; Boguta et al. 2016).

An important criterion characterizing the HAs in terms of the molecular composition and their origin is the values of absorbance of their solutions in the UV-VIS range: A_280_, A_465_, A_665_, and the coefficients of absorbance A_2/4_, A_2/6_, A_4/6_, and ΔlogK. Coefficients A_2/4_, A_2/6_, A_4/6_, and ΔlogK are important indices of the degree of advancement of the humification of organic materials and the characteristics of the HS produced, as well as changes in the properties of the HAs which occur due to various anthropogenic factors (Kumada 1987; Chin et al. 1994; Tan 1998; Gonet and Debska 1999; Chen et al. 2002; Weishaar et al. 2003; Moran Vieyra et al. 2009; Polak et al. 2011; Rodriguez et al. 2016). For example, the research of the spectrometric parameters of the HAs of soils collected from primary forests, secondary forests, coffee plantations, and cultivated lands performed by Watanabe et al. (2001) suggests that the degree of HAs humification varied from site to site.

Infrared spectroscopy (FTIR) has also been a technique used for the structural characterization of HS, especially for the identification of functional groups within the humus macromolecule (Tan 1998; Cocozza and Miano 2002; Pajaczkowska et al. 2003; Polak et al. 2011; Kukuls et al. 2019).

Some essential information on the transformation of HAs is also provided by the results of high-performance liquid chromatography. Woelki et al. (1997), Preuße et al. (2000), Banach-Szott and Debska (2006), Sierra et al. (2006), and Debska et al. (2010), using the chromatographic analysis, separated the hydrophilic (HIL) and hydrophobic fractions (HOB-1 and HOB-2). The ratios of those fractions affect the solubility of HAs and, as a result, their migration deep down the soil profile. According to Debska et al. (2007) and Debska and Gonet (2007), with an increase in the degree of humification, the share of the HIL fraction in the molecules of HAs increases and the share of HOB fractions decreases. As a result, the HAs molecules with a higher “degree of maturity” showed a higher value of the HIL/ΣHOB ratio.

The aim of the paper has been to present the land use and soil types effect on the characteristics of HAs determining the OM quality in the soil of various types. There are many results on the processes of OC stabilization by binding clay; however, some results on the changes in elemental composition and the other properties of HAs depending on the land use and soil types are missing. It was hypothesised that the effect of the land use on the properties of HAs will depend on the particle size distribution (soil types). The research has been performed in three ecosystems: agricultural, forest, and meadow, located in various parts of Slovakia. The properties of HAs have been determined from the assay of the elemental composition, spectroscopy in the UV-VIS, and IR ranges and the HIL-HOB properties applying the high-performance liquid chromatography.

## Materials and methods

### Materials

The experiment included 4 soil types: *Chernozem (Ch)*, *Luvisol (Lu)*, *Planosol (Pl)*, and *Cambisol (Ca)* (IUSS Working Group WRB 2014), each in three types of ecosystems: agri-ecosystem (AE), forest (FE), and meadow ecosystem (ME).

**Fig. 1:**
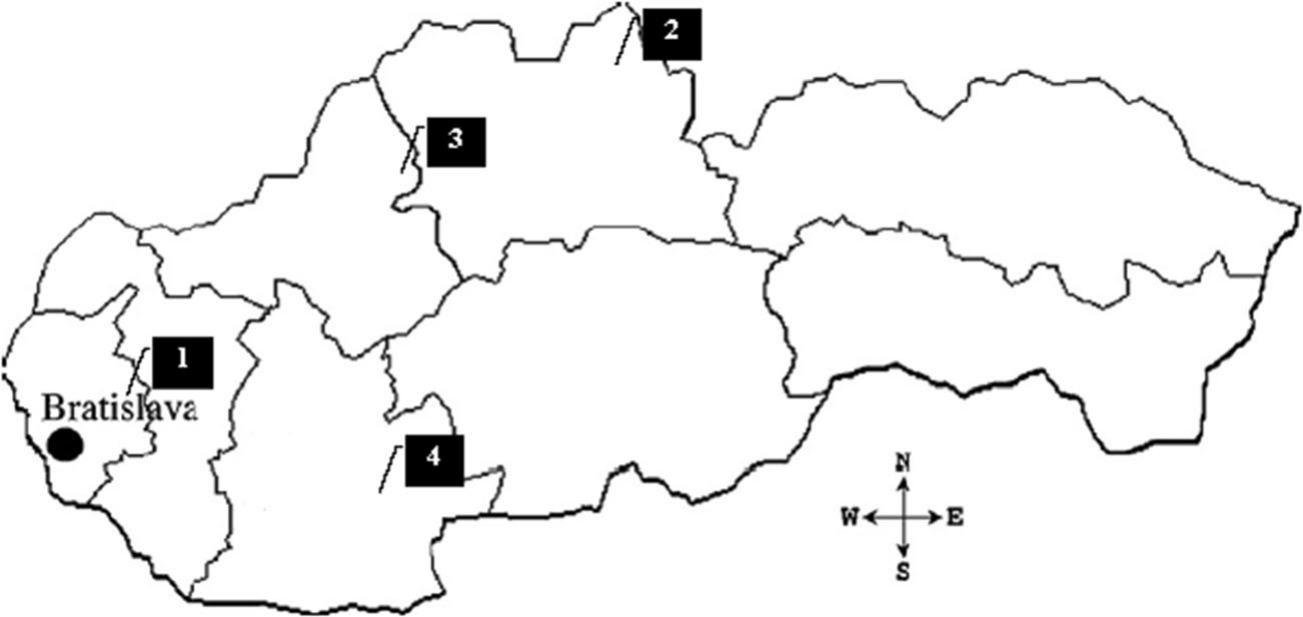
Soil sampling locations: 1 – Voderady, 2 – Vavrecka, 3 – Prietrz, 4 – Plave Vozokany

The areas are located in different parts of Slovakia (Fig. 1). *Chernozem (Ch)* comes from the location of Voderady (48°16′N, 17°34′E), found on the northern border of the Danube Basin. The geological structure shows the Neogene strata, which consist mainly of claystones, sandstones, and andesites, covered with younger quaternary rocks represented by different fluvial and Aeolian sediments (Sajgalik et al. 1986). It is located in a warm climate region with an average annual temperature of 9.6°C and the total annual precipitation of 560 mm (Korec et al. 1997).

*Planosol* (*Pl*) comes from the location of Vavrecka (49°38′N, 19°47′E), found in Horna Orava, which is part of the Flysch Belt. The geological structure shows the alternation of clay slates, sandstones, and conglomerates in the layers of various depth. It is located in a cold climate region with an average annual temperature of 4.6°C and the total annual precipitation of 1010 mm (Sajgalik et al. 1986).

*Cambisol* (*Ca*) comes from the location of Prietrz (48°40′N, 17°26′E), on the Myjava Hills. The geological structure includes the Carpathian flysch, which consists mainly of marine deposits of claystones, shales, and sandstones (Sajgalik et al. 1986). It is located in a temperate climate region with an average annual temperature of 8.0°C and the total annual precipitation of 681 mm (Korec et al. 1997).

*Luvisol (Lu)* comes from the location of Plave Vozokany (48°06′N, 18°46′E), in the northeast of the Danubian Upland. The geological structure involves clay, gravels, and sands covered with quaternary sediments (Sajgalik et al. 1986). It is located in a warm climate region with an average annual temperature of 10.4°C and the total annual precipitation of 589 mm (Korec et al. 1997).

The agri-ecosystem included four crop rotations for each soil and land use (Table 1). The fields in the agri-ecosystems were located on different farms under real production conditions. The forest ecosystems were natural forests with human control, in all the cases they were hundreds of years of age. The dominant species in the stands were on *Chernozem* - *Quercus*, on *Luvisol* - *Alnus*, on *Planosol* - *Picea*, and on *Cambisol* - *Fagus*, *Quercus*, and *Carpinus*. The meadow ecosystems were created by man at least 30 years ago.

**Table 1:** The crop rotations for soils

Soils	Vegetation	Soils	Vegetation
*Chernozem*	*Helianthus annuus*	*Planosol*	*Triticum aestivum L.*
	*Triticum aestivum* L.		*Zea mays*
	*Zea mays*		*Brassica napus var. napus*
	*Hordeum vulgare*		*Triticum aestivum L.*
	*Triticum aestivum* L.		*Zea mays*
	*Helianthus annuus*		*Zea mays*
	*Triticum aestivum* L.		*Hordeum vulgare*
	*Zea mays*		*Zea mays*
*Luvisol*	*Medicago sativa*	*Cambisol*	*Medicago sativa*
	*Helianthus annuus*		*Medicago sativa*
	*Triticum aestivum* L.		*Triticum aestivum L.*
	*Pisum sativum*		*Brassica napus var. napus*
	*Brassica napus* var*. napus*		*Triticum aestivum L.*
	*Triticum aestivum* L.		*Zea mays*
	*Helianthus annuus*		*Helianthus annuus*
	*Triticum aestivum* L.		*Triticosecale*

### Methods

The soil samples were collected with the Egner stick in three replications down to the depth of 0.30 m in the forest and meadow ecosystems. In the agri-ecosystems, collections were made in different fields with different crop rotations and different applications of farmyard manure (in 12 replications, the depth of 0.30 m). The distance between the replications was 20 m in equilateral triangle. The distance between fields differed, depending on the locations; however, it was not more than 2 km. The distance between the locations (Fig. 1) was not more than 200 km. The sample replications for chemical analyses were combined and carefully mixed, dried in room temperature, and sieved (2 mm).

#### Basic parameters of soils

The particle size distribution was determined after dissolving CaCO_3_ with 2 M HCl and the oxidation of OM-with 30% H_2_O_2_. Silt, sand, and clay fractions were assayed applying the pipette method (Van Reeuwijk 2002). In the soil samples, the TOC was assayed applying wet combustion (Orlov and Grishina 1981). The soil pH was potentiometrically measured in a supernatant suspension of a 1:2.5 soil/liquid mixture, and the liquid was 1 M KCl (pH_KCl_) (Van Reeuwijk 2002).

#### Extraction of humic acids

HAs were extracted and purified according to the following procedure (Debska et al. 2010):

- Decalcification (24 h) with 0.05 M HCl (1:10 w/v). After centrifugation the residue was rinsed with distilled water till neutral.
- The extraction (24 h) of the remaining solid with 0.5 M NaOH (1:10 w/v), with occasional mixing, followed by centrifugation.
- The precipitation (24 h) of HAs from the resulting alkaline extract with 2 M HCl to pH=2 and centrifugation.
- The purification of the resulting HAs; the HAs residue was treated with a mixture of HCl/HF (950 mL H_2_O, 5 mL HCl, 5 mL HF) over a 24-h period, followed by centrifugation. This procedure was repeated three times. The HAs residue was treated with distilled water until a zero reaction to chloride was achieved.

The preparations were lyophilised and powdered in the agate mortar. The ash content in the HAs preparations was lower than 2%.

#### Characteristics of humic acids

The HAs separated were analysed for:

- Elemental composition (Perkin Elmer, series II CHN analyser, Shelton, USA). The H/C, O/C, O/H, and N/C atomic ratios and ω (internal oxidation degree) were calculated using the following formula:

$$ \upomega =\left(2\mathrm{O}+3\mathrm{N}-\mathrm{H}\right):\mathrm{C} $$

where O, N, H, C-content in atomic %.

- UV-VIS absorption spectra (Perkin Elmer UV-VIS Spectrometer, Lambda 20, Ueberlingen, Germany). VIS spectra were obtained from 0.02% HAs solutions in 0.1 M NaOH and UV-spectra after fivefold dilution. The absorbance was measured at 280 nm (A_280_), 400 nm (A_400_), 465 nm (A_465_), 600 nm (A_600_), and 665 nm (A_665_) and used to calculate the coefficient values:
- A_2/4_ – 280 nm and 465 nm absorbance ratio
- A_2/6_ – 280 nm and 665 nm absorbance ratio
- A_4/6_ – 465 nm and 665 nm absorbance ratio
- ΔlogK = log A_400_ - log A_600_ (sKumada 1987);
- Infrared transmittance spectra (Perkin-Elmer FT-IR Spectrometer Spectrum BX, software Spectrum V2.00, Llantrisant, Great Britain) over 400–4400 cm^−1^ were recorded for HAs (3 mg) in KBr (800 mg). To increase the legibility of the spectra, deconvolution was applied, with a filter making the bands of γ = 4 narrower and using the process of smoothing, for which the length parameter was l = 80% (Cocozza and Miano 2002).
- HIL and HOB properties were determined with liquid chromatograph HPLC Series 200 with a DAD by Perkin-Elmer, Shelton, USA. The separation involved the use of column X-Terra C18, 5 μm, 250 × 4.6 mm. The solutions of HAs were applied in 0.01 M NaOH of the concentration of 2 mg mL^−1^; the injection of the sample was 10 μL; solvent – acetonitrile – water; solvents flow in the gradient (ratio H_2_O:ACN (v/v) over 0–6 min – 99.5: 0.5, 7–13 min – 70: 30, 13–20 min – 10: 90); detection – at the excitation/emission wavelength (λex/λem) of 270/330 nm. Based on the areas determined under peaks, the share of HIL and HOB (∑HOB = HOB-1 + HOB-2 + HOB-3) fractions in HAs molecules and parameter HIL/∑HOB was determined (Woelki et al. 1997; Preuße et al. 2000; Debska et al. 2007).

#### Statistical analysis

The significance of differences of the parameters between land uses (agri-ecosystem (AE), forest (FE), and meadow ecosystems (ME)) within a soil type was evaluated with Duncan’s post hoc test at p < 0.05.

The effect of the soil type and the land use on the properties of HAs was defined with cluster analysis. The method involves dividing the data set into groups to produce clusters in which the elements are similar to one another and, at the same time, different from the elements from the other groups. The groups of similar treatments are presented in a form of dendrogram. In a given group, the smaller the Euclidean distance, the more similar the objects. Data clustering was performed with the Ward method. The analysis was made after data standardization. The cluster analysis was performed based on the elemental composition (C, H, N, O, H/C, O/H, O/C, N/C, ω), spectrometric parameters (A_280_, A_465_, A_665_, A_2/4_, A_2/6_, A_4/6_, and ΔlogK), as well as the HIL-HOB properties. The method involves dividing the data set into groups to produce clusters where the elements are similar to one another and, at the same time, different from the elements of the other groups. The relationships between the share of clay, silt, and sand fraction and the basic parameters of HAs were defined using the Pearson’s correlation coefficients (P ≤ 0.05). The above relationships were determined using statistics software STATISTICA MS 12.

## Results and discussion

### Basic parameters of soils

One of the key factors controlling the rate of the SOM circulation is field aggregates produced by combining clay particles with organic particles (mineral-organic particles). The soils differed significantly in terms of the particle size distribution (Table 2). The highest content of silt and clay fractions and the lowest of the sand fraction were recorded for *Luvisol*. *Chernozem* showed a similar content of sand and silt fractions (34.9 and 36.6%, respectively). *Cambisol* demonstrated a similar content of sand and clay fraction (23.0, 24.5%, respectively) and *Planosol* a similar content of silt and clay. The lowest content of TOC was reported for *Cambisol* and the highest for *Planosol*. The analysis of correlation did not show a significant dependence between the content of clay and the content of TOC; however, as reported in literature, one cannot exclude the effect of clay on the stability of SOM (Wiesmeier et al. 2019). As evident from the literature reports (Six et al. 2002; Wiesmeier et al. 2015), it is not just the quantity of the fine fraction but also its quality which drives SOC retention in soils. Liang et al. (2009) and Wiesmeier et al. (2015) show that the correlation between SOC and clay strongly depends on climate conditions, land use, and clay type. However, as reported in literature (Wiesmeier et al. 2019), despite a lack of significant correlations, one cannot exclude the effect of clay on SOM stability.

**Table 2:** Basic characteristics of soil samples (mean values and standard deviation)

Soils	Ecosystems	Sand	Silt	Clay	pH/KCl	TOC
		(%)				(g kg^−1^)
Ch^1^	AE	29.8±2.0^2^	40.4±1.1	29.8±2.0	7.28	17.9±1.7
	FE	38.3±1.0	33.0±1.9	28.7±1.0	7.52	29.6±1.1
	ME	36.7±0.9	36.3±0.6	27.0±1.1	7.57	29.1±1.0
Mean		34.9	36.6	28.5		25.5
Lu	AE	13.2±0.8	58.0±0.8	28.8±0.3	5.85	14.8±1.9
	FE	19.1±0.9	52.1±1.8	28.8±2.3	5.05	30.1±0.9
	ME	14.5±1.0	51.5±1.1	34.0±2.1	6.56	18.9±0.6
Mean		15.6	53.9	30.5		21.3
Pl	AE	63.9±0.7	14.0±0.7	22.1±0.3	5.97	20.9±1.9
	FE	45.9±1.1	37.8±0.3	16.3±1.1	5.01	36.9±0.3
	ME	60.4±0.8	17.9±2.2	21.7±1.4	6.01	22.5±1.0
Mean		56.7	23.2	20.0		26.8
Ca	AE	34.9±0.6	44.3±1.0	20.8±1.2	7.08	13.0±1.0
	FE	17.0±1.0	58.0±1.3	25.0±1.0	6.37	16.7±0.4
	ME	17.1±0.6	55.1±0.9	27.8±1.5	5.93	11.6±0.7
Mean		23.0	52.5	24.5		13.8

^1^*Ch Chernozem*, *Lu Luvisol*, *Pl Planosol*, *Ca Cambisol*, *AE* agricultural ecosystem, *FE* forest ecosystem, *ME* meadow ecosystem, *TOC* total organic carbon

^2^Standard deviation (n=3)

### Properties of humic acids

According to De Moraes et al. (2011), the changes in land use practices can alter the chemical properties of soil HS. In this paper, the changes in land use practices are clearly reflected in the elemental composition (in atomic %) of HAs (Table 3). The content of C in the molecules of HAs ranged from 33.38 to 39.55%, H from 35.57 to 43.81%, N from 2.55 to 4.21%, and O accounted for 18.10–22.80%. The highest share of C was recorded for the molecules of the HAs of *Luvisol* sampled from the forest ecosystem. The lowest share of that element as well as N was found for the HAs of *Planosol* (FE). The lowest content of H and the highest content of O were recorded in the HAs of *Chernozem* from the agri-ecosystem (AE).

**Table 3:** Mean values of elemental composition with standard deviation and atomic ratio of humic acids

Soils	Ecosystems	C	H	N	O	H/C	N/C	O/C	O/H	ω^4^
		[% atomic]								
Ch^1^	AE	38.93±0.29^3^	35.57±0.38	2.71±0.04	22.80±0.18	0.91b^2^	0.070c	0.586a	0.641a	0.466a
	FE	34.57±0.06	43.13±0.10	4.21±0.03	18.10±0.05	1.25a	0.122a	0.524c	0.420c	0.164c
	ME	35.97±0.13	40.27±0.26	3.79±0.09	19.96±0.19	1.12a	0.105b	0.555b	0.496b	0.307b
Lu	AE	37.74±0.06	38.21±0.21	2.67±0.19	21.37±0.30	1.01a	0.071a	0.566a	0.559a	0.333a
	FE	39.55±0.68	37.23±0.92	2.92±0.07	20.30±0.61	0.94ab	0.074a	0.513c	0.545ab	0.307b
	ME	37.71±0.64	38.73±0.19	2.90±0.05	20.66±0.85	1.03a	0.077a	0.548b	0.533b	0.300b
Pl	AE	33.81±0.27	43.81±0.69	2.76±0.08	19.62±0.33	1.30a	0.082a	0.580b	0.448c	0.110c
	FE	33.38±0.43	43.65±0.35	2.55±0.13	20.42±0.56	1.31a	0.076b	0.612a	0.468b	0.145b
	ME	34.31±0.25	41.59±0.43	2.93±0.08	21.17±0.12	1.21ab	0.085a	0.617a	0.509a	0.278a
Ca	AE	34.09±0.75	43.63±0.75	3.77±0.14	18.52±0.21	1.28a	0.111a	0.543ab	0.424c	0.138c
	FE	35.40±0.51	41.77±0.12	3.26±0.12	19.59±0.24	1.18ab	0.092b	0.554a	0.469b	0.203b
	ME	36.60±0.78	40.2±0.72	3.15±0.05	20.10±0.85	1.10b	0.086b	0.551a	0.502a	0.262a

^1^*Ch Chernozem*, *Lu Luvisol*, *Pl Planosol*, *Ca Cambisol*, *AE* agricultural ecosystem, *FE* forest ecosystem, *ME* meadow ecosystem

^2^Values followed by a lowercase letter are not significantly different at 5%

^3^Standard deviation (n=3)

^4^ω - internal oxidation degree

Table 3 also presents the values of the C, H, O, and N atomic ratios. With the values of H/C atomic ratio, one can determine the degree of condensation of aromatic rings and, as a result, the “degree of maturity” of the molecules of HAs. The values of the H/C ratio for all the variants ranged from 0.91 to 1.31 (Table 3), which shows that the HAs analysed contained the aromatic systems coupled with the aliphatic chain containing up to 10 atoms of C (Van Krevelen 1950). The parameter used to describe the advancement of the process of humification is also the degree of internal oxidation (ω) of the HAs molecules and the O/C, O/H, and N/C ratios. Higher ω, O/C, and O/H values and lower H/C values correspond to the HAs with a higher “degree of maturity” (Sanchez-Monedero et al. 2002; Gonet et al. 2007).

Dergacheva et al. (2012) stress that the value of the H/C ratio depends on the conditions the HAs originated from. As seen from the data presented (Table 3), the direction of the changes in the elemental composition of HAs molecules was conditioned by the soil type. The lowest values of the H/C and N/C ratios as well as the highest values of the O/H ratio and the highest ω value were recorded for the molecules of the HAs of *Chernozem* in the agri-ecosystem, while the lowest values of the O/H ratio and the highest of the N/C ratio were found for the HAs of *Chernozem* of the forest ecosystem. The above dependencies point to the HAs of agri-ecosystem showing the highest and the HAs of the forest ecosystem—the lowest “degree of maturity” (Sanchez-Monedero et al. 2002; Gonet et al. 2007).

Of the HAs of *Luvisol* the highest content of C and the lowest of H were noted for the HAs of the forest soil, whereas the HAs of the arable soil showed the highest value of ω and the O/C ratio. The HAs quality parameters in meadow soil, in general, did not demonstrate any significant differences, as compared with the molecules of the HAs of forest ecosystems.

Of the HAs of *Planosol*, the highest content of C and the lowest content of H and, as a consequence, the lowest value of the H/C ratio and the highest values of O/H and ω were recorded for the HAs of meadow soil. The HAs of the agri-ecosystem showed the lowest value of the O/C ratio.

As for the HAs of *Cambisol*, the lowest content of C and O and the highest content of H and the highest value of the H/C ratio and the lowest values of the O/H ratio and ω were noted for the HAs of the arable soil. Interestingly, the significant differences were reported in the value of the O/H ratio and parameter ω between the HAs of the soils of all the ecosystems investigated.

**Fig. 2:**
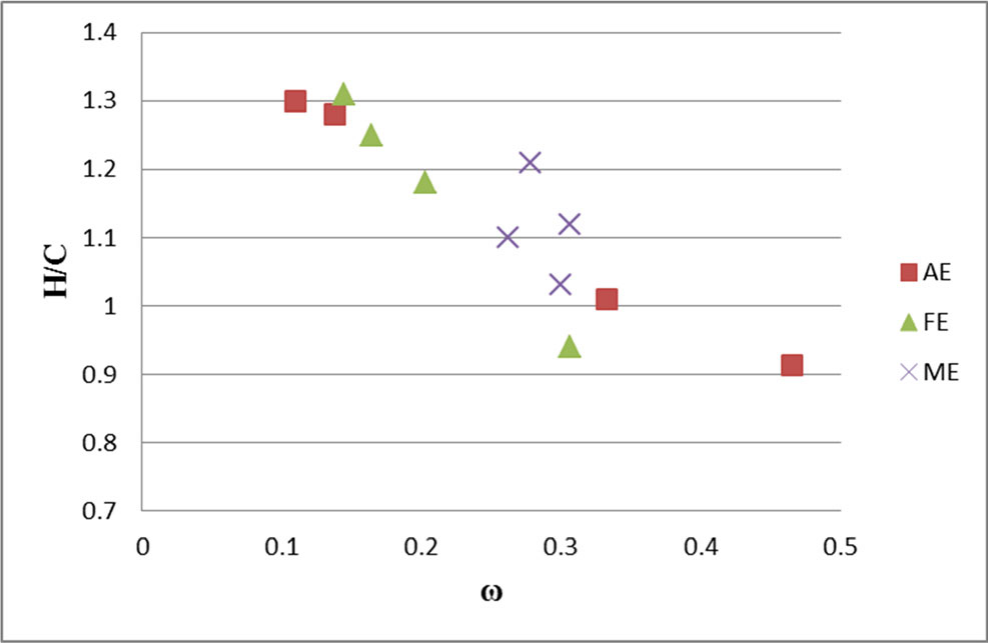
Relationship between the H/C atomic ratio values and parameter ω, where H, C – content in atomic %, ω – internal oxidation degree, AE – agri-ecosystem, FE – forest ecosystem, ME – meadow ecosystem

The impact of the land use on the properties of HAs is confirmed by the diagram of the relationships between the values of the H/C atomic ratio and parameter ω (Fig. 2). The molecules of the HAs of meadow soils showed similar values irrespective of the soil type. As for the HAs of the other ecosystems, that soil type was an additional factor determining their properties (e.g. HAs of *Luvisol*). Generally, the HAs of *Luvisol* showed the highest contents of C and lower contents of H, as compared with the HAs of the other soils (Table 3). The highest C content in the HAs of *Luvisol* of the forest ecosystem resulted in a low content of the H/C ratio and a high value of ω, as compared with the HAs of the forest ecosystem, of the other soil types.

The existing research results have indicated some dependencies between the values of absorbance at the wavelength of 280, 465, and 665 nm and the structure of HAs. The lowest values of absorbance coefficients were found for the HAs of *Luvisol* irrespective of the land use and for the molecules of the HAs of *Chernozem* sampled from the agri-ecosystem (Table 4). As reported by, e.g., Kumada (1987), Howard et al. (1998), Tinoco et al. (2015), and Filcheva et al. (2018), lower values of absorbance and higher values of the following coefficients, A_2/4_, A_2/6_, A_4/6_, and ΔlogK, point to a chemical “young age” of HAs. Young humic acids show a lower degree of condensation of aromatic structures and a lower molecular weight, as compared with the HAs with a high degree of humification. As results from the data presented in Table 4, the HAs differed in the degree of maturity. Of all the HAs of *Luvisol*, higher values of the coefficients of absorbance were recorded for the HAs of meadow soil, as compared with the HAs of forest soil and under agricultural use. Similarly, for the HAs of meadow soil of *Planosol*, there were found higher values of A_2/6_ and ΔlogK, as compared with the HAs of the agri-ecosystems. However, the HAs of *Cambisol* under meadow ecosystem use showed, in general, lower values of the coefficients of absorbance as compared with the HAs of the other variants. The values of the coefficients of absorbance recorded for the HAs of *Cambisols* confirm the dependencies reported by Jonczak (2013), who for the HAs of soils under different land uses (forest, meadow, arable field, fallow, post-arable afforestation) noted a higher maturity of humus in stands with grass vegetation (meadow, fallow, afforestation with birch with dense grass cover in forest floor) in relation to other stands. Watanabe et al. (2001) have demonstrated that the relationship between the land use and the degree of HAs humification differed depending on the soil sampling site. On the one hand, the degree of humification of HAs in the topsoil was greatest under secondary forest and least under coffee plantation, whereas for the soil sampled from another site, the degree of humification of HAs was greater under agriculture than under forests. According to the authors, the increase in the degree of humification with decreasing amount of HAs due to changing land use suggests selective decomposition of the molecules or moieties of humic acids with low degrees of humification.

**Table 4:** Mean absorbance values with standard deviation and coefficients of absorbance of humic acids

Soils	Ecosystems	280 nm	400 nm	465 nm	600 nm	665 nm	A_2/4_	A_2/6_	A_4/6_	ΔlogK^4^
Ch^1^	AE	3.94±0.12^3^	2.24±0.17	1.32±0.03	0.551±0.034	0.300±0.014	2.98b^2^	13.1b	4.40b	0.609b
	FE	2.73±0.2	1.04±0.06	0.571±0.026	0.156±0.005	0.084±0.003	4.78a	32.5a	6.80a	0.824a
	ME	3.70±0.06	1.43±0.11	0.740±0.046	0.211±0.014	0.112±0.006	5.00a	33.0a	6.61a	0.831a
Lu	AE	4.39±0.30	2.03±0.15	1.26±0.05	0.490±0.046	0.276±0.013	3.48b	15.9b	4.57b	0.617b
	FE	4.50±0.09	2.04±0.14	1.20±0.09	0.497±0.024	0.276±0.016	3.75b	16.3b	4.35b	0.613b
	ME	3.90±0.13	1.61±0.13	0.960±0.061	0.350±0.022	0.191±0.007	4.06a	20.4a	5.03a	0.663a
Pl	AE	3.25±0.14	1.11±0.07	0.616±0.008	0.199±0.014	0.097±0.003	5.28a	33.5b	6.35a	0.746c
	FE	3.15±0.06	1.03±0.07	0.564±0.018	0.178±0.008	0.085±0.006	5.59a	37.1a	6.64a	0.762b
	ME	3.15±0.04	1.10±0.06	0.582±0.016	0.178±0.002	0.085±0.002	5.41a	37.1a	6.85a	0.791a
Ca	AE	2.77±0.15	0.97±0.07	0.443±0.046	0.134±0.009	0.064±0.004	6.25a	43.3a	6.92a	0.860a
	FE	3.23±0.07	1.12±0.11	0.591±0.012	0.172±0.007	0.084±0.003	5.47b	38.5b	7.04a	0.814b
	ME	3.68±0.07	1.31±0.04	0.700±0.046	0.221±0.023	0.111±0.004	5.26b	33.2c	6.31b	0.773c

^1^*Ch Chernozem*, *Lu Luvisol*, *Pl Planosol*, *Ca Cambisol*, *AE* agricultural ecosystem, *FE* forest ecosystem, *ME* meadow ecosystem

^2^Values followed by a lowercase letter are not significantly different at 5%

^3^Standard deviation (n=3)

^4^ΔlogK = log A_400_ - log A_600_

**Fig. 3:**
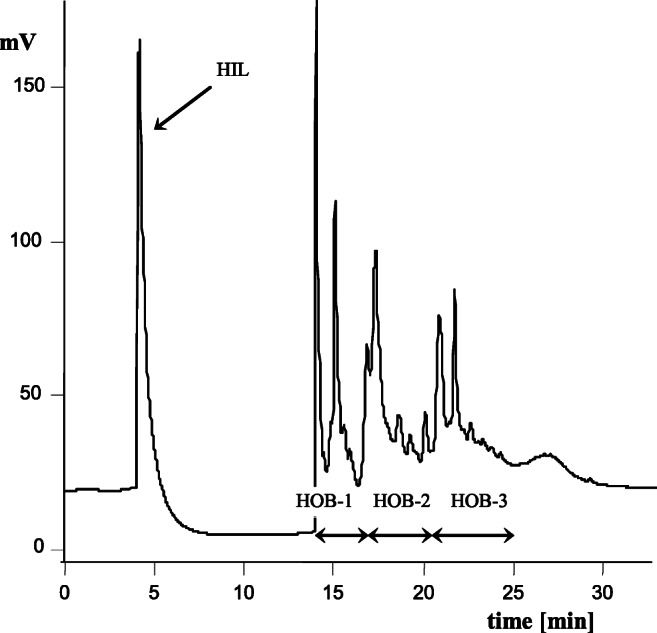
Selected RP-HPLC chromatogram of humic acids of *Chernozem* under agricultural use

HAs are built from structures with both hydrophobic and hydrophilic properties. By applying the HPLC method, one can divide the molecules of HAs into fractions which are HIL and HOB in nature (Fig. 3). The fractions of the retention time of 4.0–7.0 min show greater HIL properties, whereas the fractions of the retention time between 14.0 and 25.0 min become more and more HOB (Woelki et al. 1997; Preuße et al. 2000; Debska et al. 2007, 2010, 2012). The share of HIL fractions in the HAs molecules of the soil analysed was lower than the share of HOB fractions, and it ranged from 15.90 (*Lu* FE) to 29.46% (*Ch* FE) (Table 5). With the patterns of chromatograms for the HOB fractions, for most HAs, there were separated 3 smaller fractions: HOB-1, HOB-2, and HOB-3 (Fig. 3). Irrespective of the factors discussed (land use, soil type), of those hydrophobic fractions, in general, the HAs recorded the highest share of the HOB-2 fraction. It should be noted that the biggest differences between the shares of fractions HOB-1, HOB-2, and HOB-3 (Table 5) were identified in the molecules of the HAs of the meadow ecosystem (6.22–8.94 pp). The highest share of the ΣHOB fraction was recorded for the HAs of the forest and agri-ecosystem of *Luvisol* and the lowest for the HAs of the forest and meadow ecosystem of *Chernozem*. Of the *Chernozem* HAs, the highest share of ΣHOB was recorded for the HAs of the agri-ecosystem and of the *Planosol* HAs and the *Cambisol* HAs in the meadow ecosystem. The results point to a strong effect of the soil type on the ΣHOB properties of HAs. The changes in the share of the respective fractions resulted in changes in the value of the HIL/ΣHOB ratio. The HAs isolated from the *Luvisol*, except for ME, showed the lowest and the HAs extracted from the *Chernozem*, except for AE, the highest values of the HIL/ΣHOB ratio. The values of the HIL/ΣHOB ratio were similar for the HAs of *Cambisol* of the agri- and forest ecosystem and for the HAs of *Chernozem* of the forest and meadow ecosystem. For the HAs of *Chernozem,* no significant differences were found between the values of HIL/ΣHOB in the forest and meadow ecosystem and as for the HAs of *Planosol* between the agri- and meadow ecosystem. The share of HIL and HOB fractions and parameter HIL/ΣHOB is connected with the degree of OM humification. The values of HIL/ΣHOB of the parameter increase with an increase in the degree of maturity of HAs molecules (Woelki et al. 1997; Preuße et al. 2000; Debska et al. 2007, 2010, 2012). It should also be emphasized that the proportions of both fractions determine the solubility of HAs and, as a result, their migration deep down the soil profile (Woelki et al. 1997; Debska et al. 2007).

**Table 5:** Mean share of hydrophilic and hydrophobic fractions with standard deviation, total share of hydrophobic fractions (ΣHOB), and values of HIL/ΣHOB ratio of humic acids

Soils	Ecosystems	HIL^4^	HOB-1	HOB-2	HOB-3	∑HOB	HIL/∑HOB
Ch^1^	AE	19.74±0.44^3^	24.26±0.64	28.17±0.16	27.83±0.25	80.26a^2^	0.246b
	FE	29.46±2.23	70.54±2.15	nd	nd	70.54b	0.418a
	ME	29.19±1.75	27.24±0.77	24.77±1.00	18.80±0.57	70.81b	0.412a
Lu	AE	17.75±1.25	26.28±1.31	30.59±1.51	25.37±1.48	82.25a	0.216b
	FE	15.90±0.79	26.17±1.01	31.44±1.73	26.49±1.11	84.10a	0.189c
	ME	25.46±1.27	21.08±1.37	27.30±1.31	26.16±1.01	74.54b	0.341a
Pl	AE	23.61±1.20	24.18±1.17	28.63±1.27	23.57±1.28	76.39a	0.309b
	FE	27.53±1.56	21.01±1.60	26.02±1.41	25.44±1.15	72.47b	0.380a
	ME	22.90±1.22	25.43±1.67	29.66±1.16	22.01±0.72	77.10a	0.297b
Ca	AE	25.67±1.33	74.33±.133	nd	nd	74.33b	0.345a
	FE	25.19±1.19	24.65±1.43	28.36±1.21	21.80±1.22	74.81b	0.337a
	ME	22.17±1.4	25.83±1.71	30.47±1.16	21.53±0.93	77.83a	0.285b

^1^*Ch Chernozem*, *Lu Luvisol*, *Pl Planosol*, *Ca Cambisol*, *AE* agricultural ecosystem, *FE* forest ecosystem, *ME* meadow ecosystem

^2^Values followed by a lower-case letter are not significantly different at 5%

^3^Standard deviation (n=3)

^4^*HIL* the share of hydrophilic fraction, *HOB* the share of hydrophobic fraction

*nd* non-detectable (below detection limit)

**Fig. 4:**
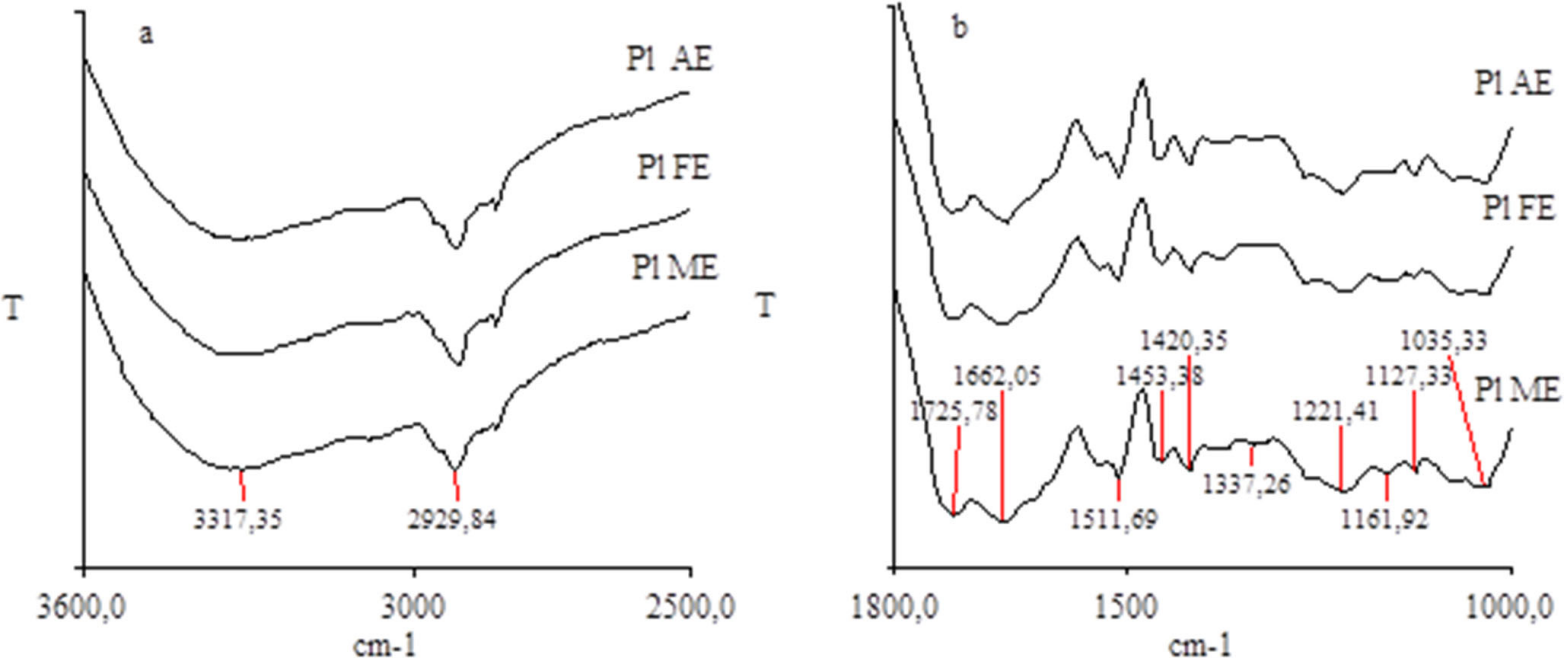
FT-IR spectra of humic acids of *Planosol (Pl)* showing the dependence of transmittance (T) on the wavenumber; **a** in the range from 3600 to 2500 cm^−1^, **b** in the range from 1800 to 1000 cm^−1^ (AE – agri-ecosystem, FE – forest ecosystem, ME – meadow ecosystem). Assignment of peaks (bands), see Table 5

Figure 4 provides sample FTIR spectra of the HAs. All the spectra were identified with a presence of the same absorption bands the ranges of which are presented in Table 6. As a result of a detailed spectral analysis, it was found that the intensity of the absorption bands in the molecules of HAs depended on the land use. The HAs of *Chernozem* and *Luvisol* under agricultural use showed a higher band intensity in the range of 1730–1710 cm^−1^, as compared with the molecules of the HAs of the forest and meadow ecosystem. The intensity of the bands in the range of 3400–3100 cm^−1^ and 2960–2920 cm^−1^ was highest for the HAs of the forest soils. The HAs of *Luvisol*, *Planosol*, and *Cambisol* of the forest ecosystem demonstrated the lowest intensity of the bands in the range of 1500 to 1000 cm^−1^. As for the HAs of *Chernozem*, the lowest band intensity for the range from 1660 to 1000 cm^−1^ was recorded for the soils under agricultural use.

**Table 6:** List of peaks (bands) present in FT-IR spectra

Wavenumber (cm^−1^)	Assignment^1^
3400–3100	O-H stretching of alcohols, phenols and acids, N-H stretching
3100–3000	C-H groups of aromatic and alicyclic compounds
2960–2920; 2850	asymmetric and symmetric C-H stretching of CH_3_ and CH_2_ group
1730–1710	C = O stretching of carboxyl, aldehyde, ketone group
1660–1620	C = O of stretching of amide groups; N-H deformation
1610–1600	C – C stretching of aromatic rings
1550–1530	N-H deformation, C = N stretching (amide II bands)
1520–1500	C-C stretching of aromatic rings
1460–1440	C-H asymmetric of CH_3_ and CH_2_
1420–1400	C-O stretching and OH deformation of phenols
1380–1320	C-N aromatic amine, COO-, C-H stretching
1280–1200	C-O stretching of aryl ethers, esters and phenols
1160–1030	C-O stretching alcohols, ethers and polysaccharides

^1^Enev et al. 2014; Zhang et al. 2017; Hayes and Swift 2018

**Fig. 5:**
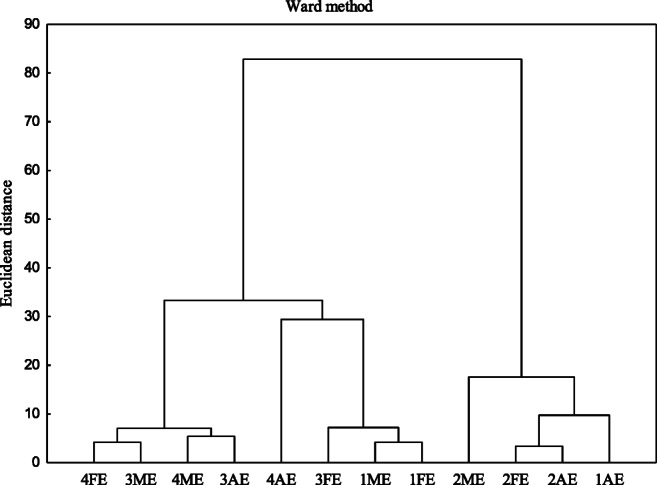
Cluster analysis determined based on humic acid parameters (1 – *Chernozem*, 2 – *Luvisol*, 3 – *Planosol*, 4 – *Cambisol*, AE – agri-ecosystem, FE – forest ecosystem, ME – meadow ecosystem)

To acquire complete information on the differences (similarities) in the chemical composition of HAs, depending on the soil type and the land use, the cluster analysis was applied based on the elemental composition, spectrometric parameters, as well as the HIL-HOB properties dividing the HAs into two groups (Fig. 5). In the first one, two subgroups were identified. The first subgroup included mostly the HAs of the meadow ecosystem of the following soil types: *Planosol* and *Cambisol* as well as the HAs of the forest ecosystem of *Cambisol* and HAs *Planosol* under agricultural use. In the second subgroup, the most similar properties were found for the molecules of the HAs of *Chernozem* in the forest and meadow ecosystem. In the second group, similar properties were noted for the HAs isolated from *Luvisol*, irrespective of the ecosystem, and the HAs extracted from *Chernozem* sampled from the agri-ecosystem. As seen from the diagram, both the soil type and the land use are the factor which determines the properties of HAs. With some approximation, one can assume that *Luvisol* is the type of the soil which is modified even least considerably by the land use.

To verify the hypothesis formulated in the Introduction, Table 7 presents the correlation coefficients (significant for p ≤ 0.05) between the particle size distribution and the properties of HAs. The HAs with a higher “degree of maturity” (a negative significant correlation between the clay content and content of H in HAs, the values of H/C, A_2/4_, A_2/6_, A_4/6_ and positive between the content of C and parameter ω and the values of absorbance) are characteristic for the soils with a higher clay fraction content and a lower sand fraction content. Even earlier Jindaluang et al. (2013) noted the effect of the particle size composition, especially the content of the clay fraction, on the content and the properties of SOM. As seen from the research, the particle size distribution, especially the content of the clay fraction, is the factor affecting also the properties of HAs, mainly their elemental composition and spectrometric properties in the UV-VIS range.

**Table 7:** Correlations between the particle size distribution content (%) and basic parameters of humic acids

	C	H	H/C	ω	A_280_	A_665_	A_2/4_	A_2/6_	A_4/6_
Clay	0.790^*^	−0.728^*^	−0.745^*^	0.642^*^	0.597^*^	0.620^*^	−0.733^*^	−0.719^*^	−0.631^*^
Silt	0.579^*^	−0.469	−0.538^*^	0.321	0.498	0.406	−0.305	−0.335	−0.378
Sand	−0.760^*^	0.597^*^	0.667^*^	−0.448	−0.585^*^	−0.514	0.481	0.481	0.493

^*^Significant correlations for p ≤ 0.05

## Conclusions

From H/C, O/C, O/H, and ω parameters and absorbance coefficients and the FT-IR spectra, it has been found that the HAs of *Chernozem* and *Luvisol* in the agri-ecosystem show a higher “degree of maturity”, as compared with the HAs of the meadow and forest ecosystem. However, for the HAs of *Cambisol*, a higher “degree of maturity” was demonstrated for the meadow ecosystem, as compared with the HAs of the agri- and forest ecosystem.

The research results have demonstrated unambiguously that the properties of HAs can be modified by the land use and the scope and that the direction of changes depends on the soil type. The research has identified that the content of clay affects the properties of HAs. Soils with a higher content of clay fraction include HAs with a higher “degree of maturity”. It is seen from the positive (significant) values of the coefficient of correlation between the clay fraction content and the content of C in the molecules of HAs, the values of the degree of internal oxidation and absorbance values, and the negative (significant) correlations between the content of the clay fraction and the content of H and the values of the H/C, A_2/4_, A_2/6_, and A_4/6_ ratios.

## Supplementary Information

ESM 1 (DOC 61 kb)
