# Supplementary material for: Properties of humic acids depending on the land use in different parts of Slovakia
Source: Environ Sci Pollut Res Int. 2021 Jun 8;28(41):58068–80. doi: 10.1007/s11356-021-14616-9 (PMC8536593; doi:10.1007/s11356-021-14616-9)
Supplement: Supplementary file 1 — (DOC 61 kb) [file 11356_2021_14616_MOESM1_ESM.doc]

**Properties of humic acids depending on the land use in different parts of Slovakia**

**Environmental Science and Pollution Research**

Magdalena Banach-Szott, Bozena Debska, Erika Tobiasova

University of Science and Technology, Department of Biogeochemistry and Soil Science, 6 Bernardynska St., 85-029 Bydgoszcz, Poland

Magdalena Banach-Szott: [mbanach@utp.edu.pl](mailto:mbanach@utp.edu.pl)


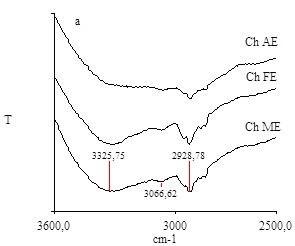

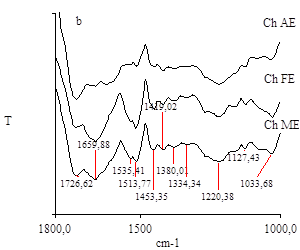


**Fig. 1.** FT-IR spectra of d humic acids of *Chernozem (Ch)* showing the dependence of transmittance (T) on the wavenumber; a – in the range from 3600 to 2500 cm^-1^, b – in the range from 1800 to 1000 cm^-1^ (AE - agri-ecosystem, FE - forest ecosystem, ME - meadow ecosystem)


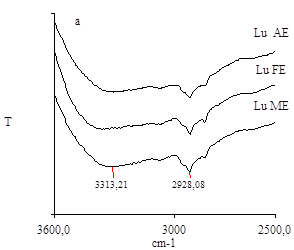

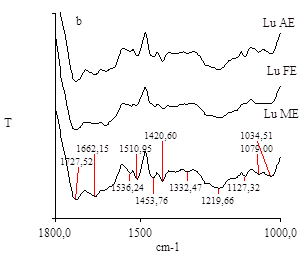


**Fig. 2.** FT-IR spectra of d humic acids of *Luvisol (Lu)* showing the dependence of transmittance (T) on the wavenumber; a – in the range from 3600 to 2500 cm^-1^, b – in the range from 1800 to 1000 cm^-1^ (AE - agri-ecosystem, FE - forest ecosystem, ME - meadow ecosystem)


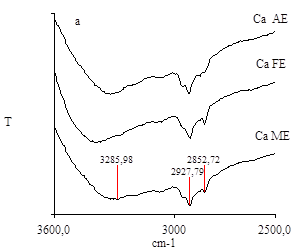

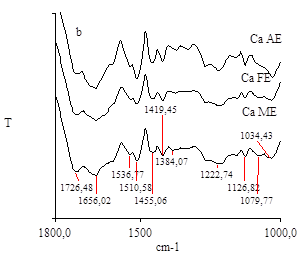


**Fig. 3.** FT-IR spectra of d humic acids of *Cambisol (Ca)* showing the dependence of transmittance (T) on the wavenumber; a – in the range from 3600 to 2500 cm^-1^, b – in the range from 1800 to 1000 cm^-1^ (AE - agri-ecosystem, FE - forest ecosystem, ME - meadow ecosystem)
